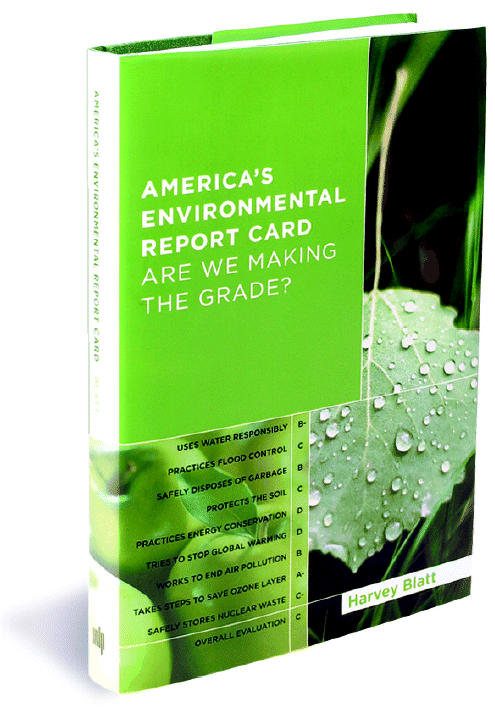# America’s Environmental Report Card: Are We Making the Grade?

**Published:** 2005-04

**Authors:** Terri Damstra

**Affiliations:** Terri Damstra is a senior scientist with the World Health Organization’s International Programme on Chemical Safety. She has worked in international environmental health, risk assessment, and toxicology for over 30 years. She is located in Research Triangle Park, NC.

By Harvey Blatt

Cambridge, MA:MIT Press, 2005. 277 pp. ISBN: 0-262-02572-8, $27.95 cloth

Protecting the environment continues to be a top priority for most Americans. Some examples of major environmental concerns include water and air pollution, hazardous waste disposal, use of toxic chemicals, and environmental threats to children. In *America’s Environmental Report Card: Are We Making the Grade?* Harvey Blatt provides a comprehensive status report on the following nine selected complex environmental problems: water pollution, dangers of floods, leaching of garbage from landfills, pesticide runoff, depletion of energy resources, global warming, air pollution, ozone depletion, and radiation from nuclear power plants and the storage of nuclear wastes. He also describes whether the situation with respect to these issues is deteriorating or improving and what actions can be taken at the individual, corporate, and political levels to ensure safe and adequate resources for future generations.

This book contains a large amount of information and statistics, often presented in charts, figures, and tables. It is easily comprehensible, and one does not “get lost in the numbers.” Its conversational tone, interspersed with anecdotes and humor, makes the 238 pages of text easy to read and at times entertaining.

However, the book does not provide a scientific review or analysis of the selected environmental issues and at times does not provide a balanced scientific viewpoint. For example, the introductory statement that “data suggesting that toxic agents in the environment have reduced the average male sperm count by 42 percent in the past 50 years” is not backed up by references nor is there mention of other references that do not support this hypothesis. Such a statement in isolation could be very alarming to the general public. The references cited in the book are mainly secondary sources, in many instances from magazine and newspaper articles. Perhaps it is this reliance on secondary sources that sometimes leads to inaccuracies and misleading statements. For example, there is no scientific evidence to indicate that global warming may result in an “epidemic” of an increase in male babies over female (p. 148). Similarly, respiratory disease is not the greatest killer of children on the planet (p. 155); diarrheal and infectious diseases are the major contributors to global child mortality.

The author, perhaps unintentionally, provides somewhat of a doomsday scenario for a number of the environmental problems covered. For example, at the beginning of the chapter of air pollution (Chapter 7) the reader is inundated with detailed descriptions and disturbing statistics on the morbidity and mortality resulting from exposure to air pollutants. Only at the end of the chapter is there a short paragraph noting that “air quality has improved markedly … since the passage of the Clean Air Act in 1972” (p. 175). Similar observations can be made for several of the other chapters.

Unfortunately, the title of this book is misleading. On the book jacket, actual grades (e.g., B, C) are given to the nine issues covered in the text. I had anticipated finding out how these grades were derived—what criteria and standards were used. Nowhere in the text is there any discussion of a “report card” or how the grades on the book jacket were determined. A concluding paragraph for each chapter provides a limited subjective evaluation on the current status of the environmental issue discussed but does not address whether America is making the grade in any systematic, objective manner.

Nevertheless, those wishing to learn about the very real environmental problems facing the United States will find Blatt’s book very interesting, full of factual information, and eminently readable. It should serve as a valuable resource for the public, government officials, and scientists new to the field. It would also be excellent background reading for a graduate course in environmental sciences.

## Figures and Tables

**Figure:**